# An Investigation to Validate the Grammar and Phonology Screening (GAPS) Test to Identify Children with Specific Language Impairment

**DOI:** 10.1371/journal.pone.0022432

**Published:** 2011-07-28

**Authors:** Heather K. J. van der Lely, Elisabeth Payne, Alastair McClelland

**Affiliations:** 1 Laboratoire de Neuropsychologie Interventionnelle, Department Études Cognitive, École Normale Supérieure, Paris, France; 2 Psychological Sciences Research Institute, Université Catholique de Louvain, Louvain-la-Neuve, Belgium; 3 Department of Psychology, Harvard University, Cambridge, Massachusetts, United States of America; 4 Department of Psychology and Clinical Language Sciences, University of Reading, Reading, United Kingdom; 5 Division of Psychology and Language Sciences, University College London, London, United Kingdom; Harvard University, United States of America

## Abstract

**Background:**

The extraordinarily high incidence of grammatical language impairments in developmental disorders suggests that this uniquely human cognitive function is “fragile”. Yet our understanding of the neurobiology of grammatical impairments is limited. Furthermore, there is no “gold-standard” to identify grammatical impairments and routine screening is not undertaken. An accurate screening test to identify grammatical abilities would serve the research, health and education communities, further our understanding of developmental disorders, and identify children who need remediation, many of whom are currently un-diagnosed. A potential realistic screening tool that could be widely administered is the Grammar and Phonology Screening (GAPS) test – a 10 minute test that can be administered by professionals and non-professionals alike. Here we provide a further step in evaluating the validity and accuracy (sensitivity and specificity) of the GAPS test in identifying children who have Specific Language Impairment (SLI).

**Methods and Findings:**

We tested three groups of children; two groups aged 3;6–6:6, a typically developing (n = 30) group, and a group diagnosed with SLI: (n = 11) (Young (Y)-SLI), and a further group aged 6;9–8;11 with SLI (Older (O)-SLI) (n = 10) who were above the test age norms. We employed a battery of language assessments including the GAPS test to assess the children's language abilities. For Y-SLI children, analyses revealed a sensitivity and specificity at the 5^th^ and 10^th^ percentile of 1.00 and 0.98, respectively, and for O-SLI children at the 10^th^ and 15^th^ percentile .83 and .90, respectively.

**Conclusions:**

The findings reveal that the GAPS is highly accurate in identifying impaired vs. non-impaired children up to 6;8 years, and has moderate-to-high accuracy up to 9 years. The results indicate that GAPS is a realistic tool for the early identification of grammatical abilities and impairment in young children. A larger investigation is warranted in children with SLI and other developmental disorders.

## Introduction

The role of language and communication is well recognized as central to education and life-long learning. Thus, children are expected to be competent users of language by the time they start school and oral language abilities are the foundation on which literacy skills develop. Yet, approximately two children in every classroom (7%), who are otherwise developing normally, experience specific and persistent language impairment; i.e., ‘specific language impairment’ (SLI). Central to these impairments for many are problems with components of grammar [1], [2]: i.e., phonology – the rules for combining sounds into word; morphology – the rules for combining words and parts of words into bigger words; and syntax – the rules determining the structural relations between words in sentences. Such grammatical deficits frequency co-occur with other developmental disorders and are found in around 50% or more of children with Autistic Spectrum Disorder, Dyslexia, and Down's syndrome [2], [3], [4], [5], [6], [7]. In cases where grammatical skills are not mastered, children are at a disadvantage at the outset [8] and it is well documented that a high percentage of pre-school children with SLI go on to experience difficulties throughout childhood and into adulthood [2], [9], [10]. In teenagers, literacy attainment was a significant factor predicting performance levels, even when the effects of non-verbal ability were removed [11], and language and literacy skills were more important in independent living than non-verbal ability [12]. Effects on emotional development [13] and mental health [8] illustrate that the impact is wider than merely language and literacy.

It is not only the individual and their family who are affected. Special education of children with persistent language impairments has significant financial implications [14] and the association between low language and literacy attainment and criminal activity further compounds the cost to society as a whole [14], [15], [16], [17], [18]. A UK government report estimated the cost of untreated language impairments to be £25 billion over a life cycle [19]. It is therefore in any nation's interest to improve outcomes for these populations [19]. Given these facts, it is all the more surprising that our understanding of the neurobiological of grammatical impairments is relatively limited; little research money is dedicated to scientific enquiry in this field [20], and many children with grammatical impairments may go undetected. Scientifically, a quick and accurate measure of cognitive performance of this uniquely human trait — grammar, would be of considerable value to geneticists, neuroscientists and cognitive scientists involved in the study of developmental disorders (e.g., SLI, Autism spectrum disorder, dyslexia, Down's syndrome) as these children frequently exhibit grammatical deficits. Clinically, identifying children in the pre-school and early school years is critical for successful remediation of language delay and/or disorder [14], [21] and therefore highly desirable. The implications of test results, however, brings an ethical dimension to language testing because of the potential effect that results may have on the lives of individuals [22]. This requires test developers to ensure that their tests are fair; however, evidence of the process is often not provided [23]. Here we provide a further step in evaluating one such test that fills the criteria of a screening test for Grammar—The Grammar and Phonology Screening (GAPS) test [24].

The GAPS test [24] was designed as a quick (10 minute) screening tool which can be administered by a professional or non-professional (including a parent). It was standardized on 668 children from across the UK, between three years four months and six years eight months of age [25]. Performance on the GAPS was significantly correlated with other standardized measures of language ability [25] providing initial data on the validity of the GAPS test. These results demonstrated that the GAPS test has good/very good internal consistency. The results also revealed that although the population tested represented a variety of demographic regions across the UK, socio-economic status did not impact on children's performance. Thus, the percentage of impaired individuals in the poorest inner city UK regions was the same as affluent regions [25]. One interpretation of this finding is that grammar and phonology abilities tapped by the GAPS test are relatively less affected by environmental factors, than genetic ones. Whatever the reason, a socio-economically neutral cognitive measure is a highly desirable tool.

The importance of test validation is two-fold; it concerns the accuracy of results obtained and their subsequent implications. The accuracy of results must be demonstrated through “generating evidence to support the well-foundedness of inferences concerning trait from test scores” p1,[23]. This is a cumulative process and evidence of a test's concurrent validity can be obtained through correlation with a range of other measures [26]. Note, however, high correlations between assessments are insufficient to validate a tests ability to identify grammatical impairment or any particular disorder as neither assessment might, for example, identify children with SLI. A core validation requirement is often quoted as being a comparison against an established “gold-standard” to measure or diagnose the ability/disability [27]. However, no such gold-standard exists when it comes to identifying grammatical impairments or SLI, and indeed there are few standardized tests that focus on tapping grammatical abilities. Therefore, in the absence of any gold-standard, this study aims to provide further evidence of the validity of the GAPS test by evaluating its accuracy in identifying children with language impairment. We do this by testing children with known SLI and children typically developing. The key questions are:

- Is performance on the GAPS test related to that on longer, standardised assessments?
- Do children with SLI “fail” the GAPS test?
- Is the GAPS test sensitive and specific in identifying children with SLI?

SLI is identified in children who present with significantly below average language ability, yet normal non-verbal abilities (IQ >80) [9], and an absence of other factors that might account for their language difficulties, such as hearing impairment, neurological dysfunction, or impairment of psycho-social abilities [9], [28]. SLI heterogeneously affects grammatical [9], [10], [29], [30], [31] and non-grammatical (lexicon, pragmatics) language components, causing problems in language expression and understanding. Whereas semantic and pragmatic understanding may be relative strengths [9], [32], [33], most individuals with SLI show particular impairments in grammatical components; i.e., broadly, syntax, morphology and often phonology [9], [10], [29], [30], [31], [33], Interestingly, these language areas have also been the most fruitful for discovering genetic links with phenotypic behaviours [34], [35].

The construction and development of the GAPS test was based on a linguistically and psychologically informed model of the underlying nature of SLI, in particular the Computational Grammatical Complexity hypothesis (CGC) [1], [2], [10], [35], but it is also consistent with the large body of data linguistically characterising SLI. The CGC hypothesis [1], [2], [10] proposes that the core impairments are in hierarchical structural computations, affecting processing and production of syntax, morphology and phonology [10], [35]. With respect to syntax, “complex” sentences involving ‘structural dependencies’ are impaired at the clause level (e.g., relating the wh-word in questions to the “empty” position that is normally filled in declarative sentences, *Who did Jo see __?* vs *Joe saw Paul*), leaving those within a phrase preserved such as number agreement (*He has jumped vs. They have jumped)* [1], [2], [36]. Structures typically affected at clause level are those associated with the linguistic concept of ‘movement’ [37] causing problems with assignment of whom does what to whom in a sentence (*The man was eaten by the fish*), or producing, processing or judging wh- questions (*Who did the fish eat?*) [36], [38], [39], marking tense syntactically [40], [41], [42] and understanding and producing embedded sentences [43], [44]. In addition to syntactic deficits, impairments in morphology, that also affects tense marking and processing, are well-documented [29], [45], [46], [47], [48]. These studies found qualitative differences in the way regular inflections (past tense verbs, and plural nouns) are stored in SLI. An increasing impairment in the phonological component is revealed in the repetition or processing of nonwords when the prosodic and metrical complexity increases [30], [49], [50]; thus ***dre*** *pa* (where the bolded syllable represents word stress) is relatively easy but *pa* ***drep*** is hard. All three components of grammar (syntax, morphology and phonology) are therefore unified by the CGC account, which proposes that children with SLI are impaired in their ability to construct hierarchically complex structures within each component [2], [10]. The CGC hypothesis is built on some 15 plus years of experimental research findings in the language acquisition and SLI fields and provided a theoretical foundation for the construction of GAPS test.

To accurately identify affected children, Rice [33] highlights the value of targeting dimensions of language which show high levels of sensitivity (the extent to which true cases of impairment are identified) and specificity (the extent to which normal abilities are demonstrated), rather than trying to capture all relevant language components. Such dimensions of language, or clinical markers, have been proposed based on core aspects of language impairment in SLI and dyslexia and should therefore be incorporated into processes of identification and diagnosis, more specifically in screening assessments. The Grammar and Phonology Screening (GAPS) test incorporates these core components which are probed through two elicited imitation procedures: the first tests syntax and morpho-syntax (‘grammar’) through a sentence repetition task and the second tests phonology — specifically the prosodic structure — in a non-word repetition task [30], [49]. The items for both subtests were taken from a number of longer, specific assessments that had been designed to identify structural grammatical and phonological impairments and abilities [51], [52], [53], [54] and in so doing provide a screening test which is more focused and fine-grained than other assessments used in the pre-school and early school years [55]. Further, the repetition procedure, by its very nature, captures both input (receptive) and output (expressive) processes. The GAPS test does not claim to be diagnostic *per se*, as this also requires non-verbal and other cognitive abilities to be tested, but highlights individuals with weaknesses in the development of either grammar and/or phonology; specific knowledge and abilities which are typically acquired by the age of four years. It also provides a quick measure of the normative range of abilities in these domains [25].

## Materials and Methods

Ethical approval was obtained from UCL and UCLH research ethics committee and Berkshire Research Ethics Committee, UK. We obtained informed written consent from all parents/guardians/next of kin of the children involved in the study consistent with our research ethics approval.

### Participants

Three groups of children participated in this study (n = 51). A control group of typically developing children (n = 30) and 21 children with specific language impairment who were recruited principally through UK specialist language resources. All participants with were diagnosed on the basis of assessment by speech and language therapists and educational psychologists (who were not associated with this study) according to a discrepancy between language receptive and expressive assessment scores and average non-verbal ability as assessed on a range of standardized assessments. Thus, this typically is a gap of at least 1.3SD with an IQ score of >80 IQ. All participants were primarily language impaired, with no additional diagnoses of social-pragmatic communication difficulties, syndromes or dyspraxia. Seventeen of these children were placed in specialist language resources at the time of the study. Five had been diagnosed with SLI and identified as potential candidates for specialist provision, but at the time of the study were receiving support in their local mainstream school or nursery and were being monitored by professionals. For 16 of the 21 participants with SLI, their diagnosis was supported by an official, legally binding, “Statement of Special Educational Needs”, provided by the local Educational Needs Department on the basis of written reports from experts. Further advice from other specialists (e.g., medical, social) or second opinions may have also been sought. A second opinion of the second author (a specialist Speech and Language Therapist) had been requested for a few of the children and she consequently provided an assessment and report for these children for consideration. However, she was not involved in the official statement of educational needs. The remaining children had been identified as appropriate to place in language units by Speech and Language Therapists and Educational Psychologists and were awaiting a final statement from the Education Department. One participant had been referred for assessment by an educational psychologist; however, assessment was not completed by the conclusion of the study. Our assessments revealed that virtually all the children in the SLI groups had both expressive and receptive (comprehension) language impairments. The individual raw and age-adjusted Z-scores for each test are provided Appendix S1. The sample of SLI children was split into two age groups: one group, (Y)-SLI (n = 11) consisted of children with ages within the standardization range (3;4–6;8) for which GAPS was designed and the second group, (O-SLI) consisted of children between 6;9 and 8;11 (n = 10). The O-SLI children allowed us to evaluate the validity of using GAPS in this age range as we are aware that this sometimes occurs.

The control group and the majority of the SLI group were recruited from a large mainstream primary school. The abilities of all children within the standardization age of the GAPS were discussed with class teachers. The following exclusions applied:

- English as an additional language
- Statements of special educational need
- School Action or School Action Plus of the UK special educational needs code of practice

A potential control group was therefore identified and letters of invitation were sent to all these children. A random selection was made from among those who responded. Details of the participants can be found in table 1.

**Table 1:** Participant Details for the three groups of children.

	Typically Developing Children	Y-SLI	O-SLI	Total
Girls: Boys	18:12	5:6	5:5	
**Age** Mean (y:m)Age range	5;6	5;2	7;9	
	3;7–6;8	3;9–6;6	6;9–8;11	
Nursery	5	2		**7**
Reception	5	4		**9**
Year 1	10	3		**13**
Year 2	10	2	3	**15**
Year 3			7	**7**
**Total**	**30**	**11**	**10**	**51**

All children came from the same demographic area and were also broadly matched on socio-economic status. y;m  =  years;months.

Key: y;m  =  years;months Y-SLI  =  Young SLI; O-SLI  =  Older SLI.

### Tests and materials

In addition to the GAPS test, participants were individually assessed using a range of standardised language assessments which tap areas of language considered to be clinical markers of SLI. The most similar measures to the language component abilities tested in the GAPS grammar and phonology subtests were the Recalling Sentences subtest of the Clinical Evaluation of Language Fundamentals – Pre-school second UK edition (CELF Preschool 2 UK (CELF-RS [56]); and the Children's Test of Nonword Repetition (CNRep [57], respectively. We note that CNRep is designed to also assess short-term-memory among other abilities (see [58] for discussion). Measures of language comprehension tapping many aspects of language were also obtained (described in this study as ‘general’ tests); the British Picture Vocabulary Scale, Second edition (BPVS [59]) that assesses single word understanding and the Test for Reception of Grammar, 2nd edition (TROG -2 [60]) that assesses sentence comprehension. Participants were also assessed using two measures of specific areas of language competence proposed to tap core abilities of the computational grammatical system [1], [2] which is known to be frequently impaired in SLI; The Verb Agreement and Tense Test (VATT) [53] that assesses verbal tense marking and subject-verb agreement and the Test of Active and Passive Sentences (Revised) (TAPS-R) [51] that assesses the assignment of thematic roles in reversible sentences. Literacy levels were assessed using the Basic Reading subtest of the Wechsler Objective Reading Dimensions (WORD) [61]. Table 2 summarises the battery of assessments:

**Table 2:** Summary of test battery and language components tapped by the different tests.

Grammar	Phonology	General	Literacy
CELF-RS	CNRep	TROG-2	WORD Basic reading
TAPS-R	GAPS phonology	BPVS	
VATT			
GAPS grammar			

Key: CELF-RS =  Recalling Sentences subtest of the pre-school CELF-3 [56]. TAPS-R  =  Test of active and passive sentences- Revised edition [51], VATT  =  Verb and Tense Test [53], GAPS grammar  =  Grammar and phonology screening test, grammar sub-test [24], CNRep  =  The children’s test of non-word repetition [57], GAPS phonology  =  GAPS phonological subtest [24], TROG-2  =  Test of reception of grammar-2 a test of sentence understanding [60]; BPVS  =  British picture vocabulary scales [59]; WORD  =  Weschler objective reading dimensions [61].

To ensure that there was not any experimental bias (as the tester was not blind to the status of the subjects) we recorded responses, so that they could be independently evaluated. Expressive responses were recorded and replayed on a Dell PP17L laptop using Audacity software and a Samson CO1U USB studio condenser microphone. Responses were scored off-line from the recording. Stimuli for the CNRep were played on a Coomber 393 cassette recorder, using the published cassette.

### Procedure and scoring

All testing was carried out by the second author, who is a specialist speech and language therapist. The majority of participants attended one mainstream primary school, and a number other schools in the same demographic area. Testing for most participants was carried out in a quiet room at their school and for a few in a clinical setting. In order to avoid fatigue or loss of attention and to minimise the time a child was out of the classroom, assessments were divided into two sets. Participants were tested on two separate occasions no more than one month apart. Assessments were allocated to Set A or Set B according to the length, language component and process involved (i.e., reception or expression) of each assessment (see Table 3). Each cohort of five children in each diagnostic group was randomly allocated to Set A or B as the assessments they would be administered in the first testing session, thereby counterbalancing the presentation order across and within groups.

**Table 3:** Allocation of assessments to groups A and B and time required for assessment.

Set A	Testing Time(Mins)	Set B	Testing Time(Mins)
VATT	10	TROG-2	20
CELF-RS	10–12	WORD Basic Reading	7
CNRep	7	GAPS test (both subtests)	7–10
TAPS-R	15	BPVS-2	15

The key for assessments can be found in table 2 caption.

Participants received standard instructions at the start of each assessment. Further support was given if it was evident that they had not understood the task with general prompts given if necessary. All assessments were scored according to standard instructions, and a raw score obtained. The TROG-2 was scored according to complete blocks passed. The VATT yielded two raw scores; the number of correct responses for 3rd person agreement (VATT-AGR), and the number of correct verb stems marked for past tense, including overregularizations (Tense Marked, VATT-TM).

## Results

The performance of each group on the standardised assessments is summarised in Table 4. The numbers of participants shown in brackets reflects the number of children within the standardisation age-range for the test. Table 4 shows that the control group performed at a higher level than the Y-SLI and O-SLI groups in all assessments with standard scores for the control group being generally above 1.0 SD and those for the two SLI groups below -1.0 SD. However, the Y-SLI and O-SLI groups' mean vocabulary scores were within a (low) normal limit. The SLI groups produced particularly low scores in the expressive grammar tests, reflecting their characteristic grammatical difficulties. This pattern generally held for the phonology tests and reflects the potential for phonology to be a clinical marker for language or reading impairment. However, the control group produced a poor performance on the CNRep, (mean z-score of -0.72). The CNRep, like many non-word repetition tasks, is designed to be a complex psycholinguistic task tapping a range of abilities [58], [62]. The lower performance of the control group on the CNRep may reflect these factors rather than specific weaknesses in phonological processing. This was not so for the nonwords in the GAPS phonology subtest that focuses on structural phonology and systematically varies metrical and prosodic complexity [63]. The GAPS nonwords are purposefully short to avoid taxing phonological Short-Term-Memory (STM) more than necessary. Thus, the scores for the GAPS phonology subtest may reflect phonological prosodic structure [63], rather than processing factors outside the linguistic system. Indeed, the pattern of performance on the GAPS phonology subtest matched other language scores more closely, with the average percentile of the control group being above the mean, and the Y-SLI group well below. However, the O-SLI group fell within a low normal range.

**Table 4:** Summary of results from the language test battery for the three subject groups.

	Measure		Controls N = 30	Y- SLI N = 11	O-SLI N = 10
Grammar	CELF-RS	Raw, mean (SD)	24.10 (8.19)	6.64 (6.62)	14.50 (6.85)
		Raw, range	7–37	1–25	6–29
		z-score, mean	0.36	–3.82	–2.67 (n** = **1)
	VATT – AGR (n = 20)	Raw, mean (SD)	9.63 (6.75)	1.46 (3.88)	3.50 (5.76)
		Raw, range	0–20	0–13	0–18
	VATT – TM (n = 20)	Raw, mean (SD)	7.80 (4.53)	0.55 (1.81)	2.60 (4.50)
		Raw, range	2–18	0–6	0–14
	TAPS (n = 48)	Raw, mean (SD)	32.20 (5.76)	22.82 (7.82)	28.20 (8.04)
		Raw, range	22–43	12–36	15–39
	GAPS – GRAMMAR	Raw, mean (SD)	09.67 (1.97)	2.73 (2.87)	5.70 (2.98)
		Raw, range	4–11	0–10	2–10
		Percentile rank, mean	71.03	7.18	6.60
Phonology	CNRep	Raw, mean (SD)	16.80 (6.50)	3.18 (4.26)	16.60 (6.40)
		Raw, range	2–30	0–11	7–25
		z-score, mean	–0.72 (n** = **27)	–2.53 (n** = **9)	–2.43
	GAPS - PHONOLOGY	Raw, mean (SD)	6.37 (1.50)	1.00 (2.19)	4.70 (2.36)
		Raw, range	3–8	0–7	1–8
		Percentile rank, mean	61.83	9.00	31.60
General	BPVS	Raw, mean (SD)	63.10 (12.70)	43.82 (11.44)	60.70 (10.69)
		Raw, range	41–87	19–59	48–78
		z-score, mean	0.54	–.64	–.96
	TROG-2	Raw, mean (SD)	7.30 (4.13)	3.55 (3.75)	7.50 (4.40)
		Raw, range	2–15	0–14	2–14
		z-score, mean	–0.40 (n** = **27)	–1.32 (n** = **9)	–2.15
Reading	WORD	Raw, mean (SD)	14.40 (11.40)	3.36 (3.41)	13.8 (8.97)
		Raw, range	0–41	0–11	5–36
		z-score, mean	1.16 (n** = **11)	–.63 (n** = **4)	–1.27 (n = 1)

Controls =  typically developing children; Y-SLI  =  younger SLI children within the age-range of GAPS; O-SLI  =  older SLI children who are aged 6:9 to 9 years; VATT-AG  =  VATT Agreement score; VATT-TM – VATT past tense marked verb score; TROG-2 Mean Score =  Mean number of blocks passed.

### Correlations between the GAPS and other tests of language

Partial correlations, co-varying age, were carried out to measure the strength and significance of the relationship between the GAPS test and other tests. Table 5 shows the partial correlations between all assessments in the test battery. Significant correlations were found for all scores, as would be expected in a range of assessments related to language. The results indicated that the subtests of the GAPS test correlated most strongly with tests primarily tapping the same components of language. The highest correlation was between the GAPS grammar subtest and the CELF-RS, *r*(48)  = 0.87, *p*  =  <0.01, thus accounting for 74% of the variance between the two measures. The correlation between the GAPS phonology subtest and the CNRep was also strong, *r*(48)  = 0.73, *p*  =  <0.01. In addition, there was a strong correlation between scores on the individual GAPS subtests, *r*(48)  = 0.83, *p*  =  <0.01. Correlations among tests of expressive grammar were strong, and accounted for between 37% and 64% of the variance. Weaker correlations were evident between tests tapping different components of language or language-related skills such as syntax and reading (TAPS-R and WORD: *r*(48)  = 0.41, *p*  =  <0.01).

**Table 5:** Correlation matrix showing the partial correlations (controlling for age) between all the assessments in the test battery.

		Grammar					Phonology		General		Reading
		CELF-RS	TAPS – R	VATT - TM	VATT - AGR	GAPS – Gram	CNRep	GAPS - Phon	BPVS	TROG	WORD
Grammar	CELF-RS										
	TAPS - R	0.66									
	VATT – TM	0.76	0.37								
	VATT – AGR	0.70	0.47	0.80							
	GAPS -Grammar	0.87	0.66	0.65	0.61						
Phonology	CNRep	0.69	0.55	0.48	0.40	0.76					
	GAPS – Phon	0.72	0.58	0.57	0.56	0.84	0.83				
General	BPVS	0.65	0.65	0.58	0.48	0.71	0.67	0.66			
	TROG	0.67	0.56	0.60	0.64	0.62	0.50	0.47	0.53		
Reading	WORD	0.72	0.41	0.57	0.47	0.57	0.63	0.51	0.59	0.50	

(Correlations with the two scores from the GAPS subtests are highlighted).

**Figure 1:**
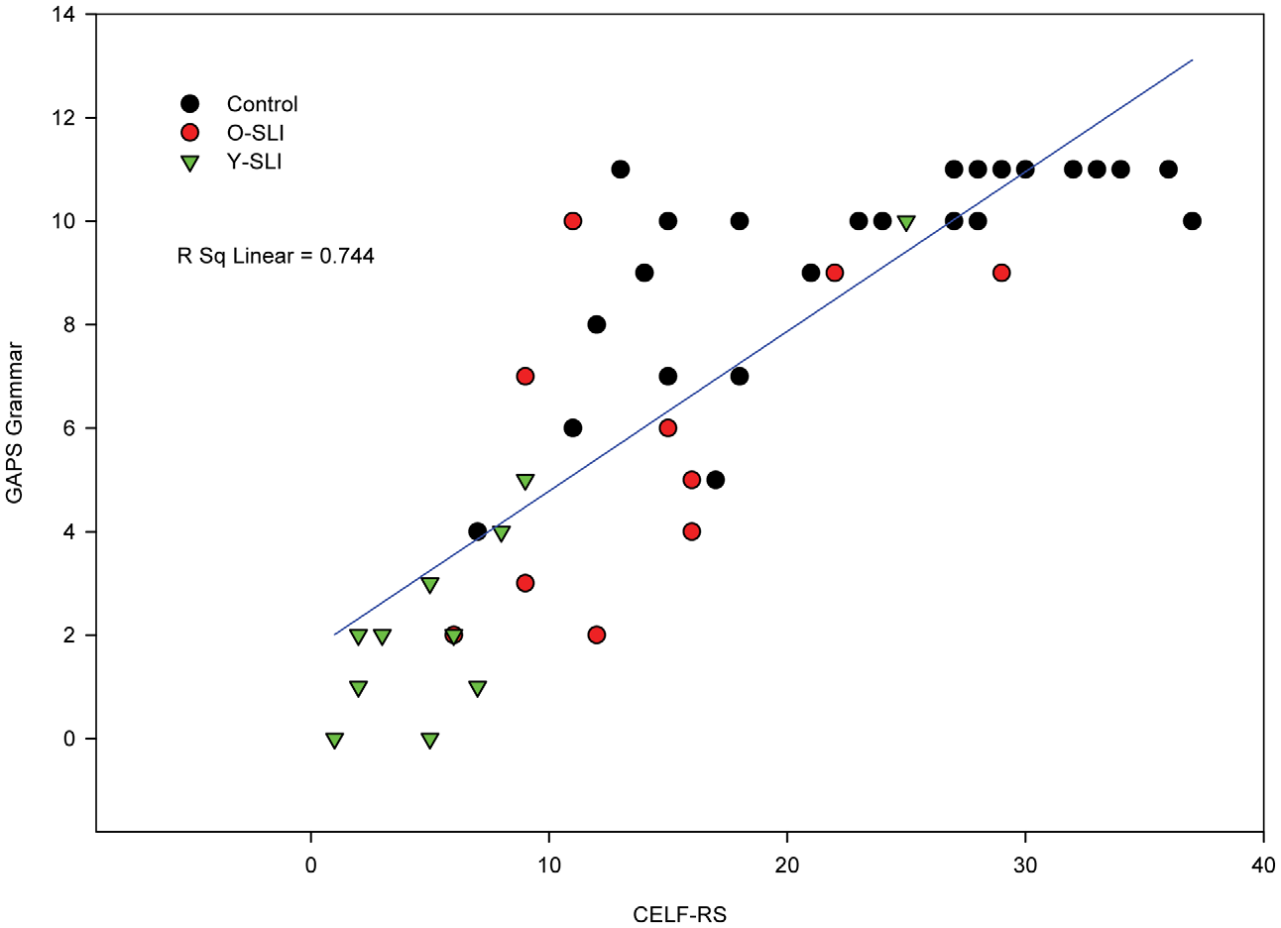
Scatterplot for the three groups' scores on grammatical tests: the GAPS grammar and the CELF-RS (CELF-Repeating Sentences) tests. The scatterplot is conducted on raw scores, so affect of age is not considered in this figure.

Scatterplots (Figures 1–3) showing individual performance of participants on the CELF-RS and GAPS-grammar, CNRep GAPS-phonology and the two subtests of the GAPS revealed that the overall strong correlations were reflected in the scores for each group of participants.

**Figure 2 pone-0022432-g002:**
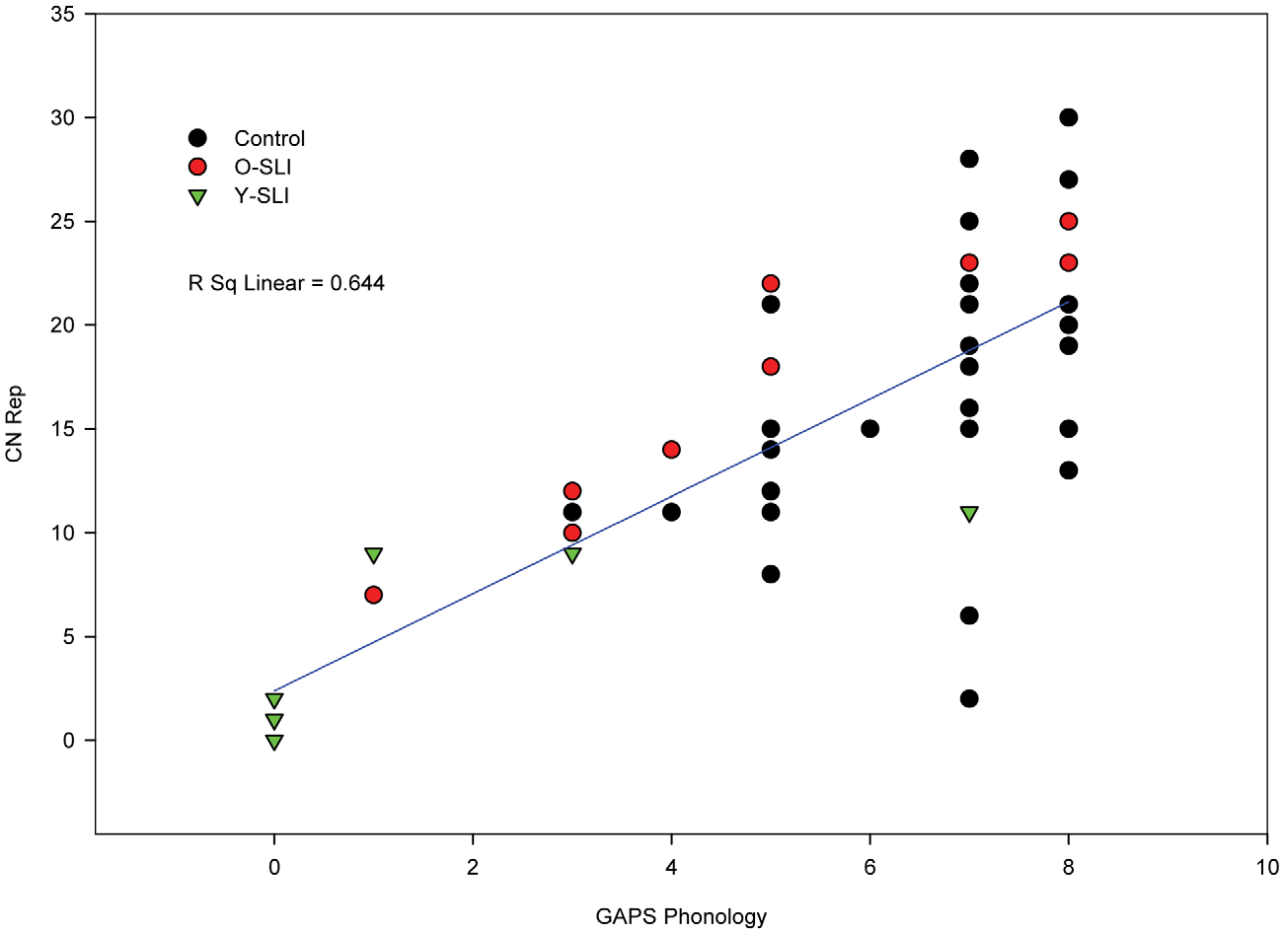
Scatterplot for the three groups' scores on phonology tests: GAPS-phonology and the CNRep tests. The scatterplot is conducted on raw scores, so affect of age is not considered in this figure.

**Figure 3:**
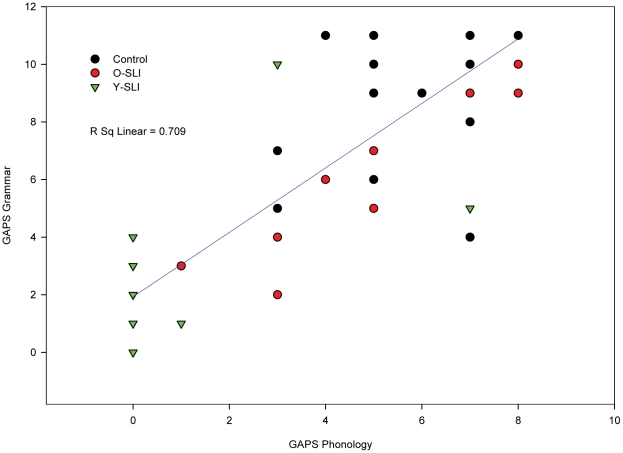
Scatterplot for the three groups' scores on the GAPS-Grammar and GAPS-Phonology subtests. The scatterplot is conducted on raw scores, so affect of age is not considered in this figure.

### Performance of the Y-SLI and O-SLI groups on the GAPS test

In order to evaluate if the GAPS test was correctly identifying children with SLI as impaired we first categorised the data into pass/fail based on the three standardised criteria provided by the GAPS manual: that is, children falling into the lowest 5^th^ percentile; the lowest 10^th^ percentile, and the lowest 15^th^ percentile which corresponds approximately to z scores of −1.64; −1.30 and −1.00; criteria that are frequently used in the literature to identify children with LI or in need of further help or support. The two groups of SLI children were considered separately: that the Y- SLI and O-SLI. Although both subtests of the GAPS are designed to be used together to identify if a child needs further assessment and has/is at risk for SLI and/or dyslexia, we will first compare the pass/fail patterns on each GAPS sub-test and compare them to the pass/fail criteria on the two standardised assessments that most closely tap the same language components. Where children were older or younger than the standardization age on the comparison test, pass/fail criteria were applied according to their performance in comparison to the nearest highest/lowest age band. Following this we evaluated the overall validity of the GAPS test by combining the pass/fail patterns on both subtests, with a fail being credited to a child if he/she failed either or both tests. Tables 6 and 7 show the pass/fail patterns on the GAPS grammar and phonology subtest respectively. Table 6 shows strong comparability between the GAPS grammar subtest and the CELF-RS. At the recommended 10% cut of point, the GAPS-Grammar subtest alone shows a moderate to high level of sensitivity with over 90% of Y-SLI children being identified. The same number of children was also identified as failing on the CELF-RS. A decrease in sensitivity is found when used with O-SLI children who are outside the test age norms. However, at the 10^th^ percentile, 70% would still be identified as failing and at the 15^th^ percentile 90% were identified. The CELF-RS at the 10^th^ percentile also identified 70% of the O-SLI as impaired and at the 15^th^ percentile criterion 90%.

**Table 6:** Numbers of Y-SLI and O-SLI children who pass/fail (%) comparing the GAPS grammar and CELF-RS at each of the three criterion: 5^th^ Percentile/-1.64, 10^th^ percentile/- 1.3; 15^th^ percentile/-1.

SLI	GAPS Grammar 5%				
**CELF-RS Z-Score -1.64**		Pass		Fail	
		Y-SLI	O-SLI	Y-SLI	OSLI
	Pass	1 (9.1)	2 (20)	0	1 (9.1)
	Fail	2 (18.2)	1 (10)	8 (72.7)	6 (60)

SLI	GAPS Grammar 10%				
**CELF-RS Z-Score -1.3**		Pass		Fail	
		Y-SLI	O-SLI	Y-SLI	OSLI
	Pass	1 (9.1)	2 (20)	0	1(10)
	Fail	0	1 (10)	10 (90.9)	6 (60)

SLI	GAPS Grammar 15%				
**CELF-RS Z-Score -1**		Pass		Fail	
		Y-SLI	O-SLI	Y-SLI	OSLI
	Pass	0	0	0	1(10)
	Fail	1 (9.1)	1 (10)	10 (90.9)	8 (80)

**Table 7:** Numbers of Y-SLI and O-SLI children who pass/fail (%) comparing the GAPS –Phonology and CNRep at each of the three criterion.

SLI	GAPS Phonology 5%				
**CNRep Z-Score -1.64**		Pass		Fail	
		Y-SLI	O-SLI	Y-SLI	OSLI
	Pass	1 (9.1)	3 (30)	0	0
	Fail	2 18.1)	3 (30)	8 (72.7	4 (40)

SLI	GAPS Phonology 10%				
**CNRep Z-Score -1.3**		Pass		Fail	
		Y-SLI	O-SLI	Y-SLI	OSLI
	Pass	1 (9.1)	1 (10)	0	0
	Fail	0	4 (40)	10 (90/9)	5 (50)

SLI	GAPS Phonology 15%				
**CNRep Z-Score -1.**		Pass		Fail	
		Y-SLI	O-SLI	Y-SLI	OSLI
	Pass	1(9.1)	1 (10)	0	0
	Fail	0	1 (10)	10 (90.9)	8 (80)

Table 7 shows the pass/fail patterns comparing the performance of the SLI groups on the GAPS phonology subtest and the CNRep. Two children were one and two months respectively below the age range for the CNRep and therefore individual scores were examined. Both children achieved a raw score below the level required for a standard score of 64 at age four years. They were therefore both judged to have failed the CNRep. For the Y-SLI, at the 10^th^ percentile, the GAPS phonology subtest and the CNRep both identified 90.5%, of the children. For the O-SLI children, however, for the GAPS phonology subtest the percentage of children failing was lower, with only 50% at the 10^th^ percentile but 80% at the 15^th^ percentile being identified. In contrast, the CNRep, which is standardized for older children, proved highly accurate in identifying the O-SLI group with 90% being identified at the 10^th^ and the 15^th^ percentiles. However, although the CNRep was not evaluated in this study as a test to identify SLI and indeed it was not designed to do so, it is noteworthy that 12 (40%) of the 30 control children would have failed the CNRep at the 15^th^ percentile.

We next turned to evaluating how well the GAPS test overall identifies children as having language impairments in grammar and/or phonology. Table 8 shows that for the Y-SLI, 63.3% failed both subtests at the 5^th^ percentile cut-off point, rising to 81.8% for the 10^th^ and 15^th^ percentile. More importantly, at the 5^th^ and 10^th^ percentiles the Y-SLI children who passed the phonology subtest (one child) failed the grammar subtest; and the three children who passed the grammar subtest, failed the phonology. Thus 100% of the children were identified by one or both subtests as having grammatical and or phonological problems, potentially SLI and in need of further assessment and help.

**Table 8:** GAPS overall accuracy: numbers of Y-SLI and O-SLI children who pass/fail (%) on the GAPS –Phonology and GAPS Grammar at each of the three criterion.

SLI	GAPS Phonology 5%				
**GAPS Grammar 5%**		Pass		Fail	
		Y-SLI	O-SLI	Y-SLI	OSLI
	Pass	0	3 (30)	3 (27.3)	0
	Fail	1 (9.1)	3 (30)	7 (63.3)	4 (40)

SLI	GAPS Phonology 10%				
**GAPS Grammar 10%**		Pass		Fail	
		Y-SLI	O-SLI	Y-SLI	OSLI
	Pass	0	3 (30)	1 (9.1)	0
	Fail	1 (9.1)	2 (20)	9 (81.8)	5 (50)

SLI	GAPS Phonology 15%				
**GAPS Grammar 15%**		Pass		Fail	
		Y-SLI	O-SLI	Y-SLI	OSLI
	Pass	0	1 (10)	1 (9.1)	0
	Fail	1 (9.1)	4 (40)	9 (81.8)	5 (50)

The ability of GAPS to identify children who may need help at older ages, up to 8;11 in this sample, is reduced, but the test still remains moderately to highly accurate. At the 10^th^ percentile, 70% of the sample was identified as failing one or both tests, making it moderately accurate. However this rose to 90% at the 15^th^ percentile cut off. Only one child in the O-SLI group passed both subtests at the 15^th^ percentile. However, it is evident from Table 8 that fewer children fail both subtests with only 50% of the children failing both grammar and phonology. Interestingly, of the remaining children, 40% failed the grammar subtest at the 15^th^ percentile, but passed the phonology. We return to this point in the discussion.

### Sensitivity and Specificity of the GAPS test

The pass/fail patterns of the Y-SLI group (see Table 8) indicated that all of the clinical population within the age range of GAPS, and most of the O-SLI at the higher cut off criterion, indeed failed one or both subtests of the GAPS test. Table 9 shows the pass/fail patterns of the control group comparing both subtests of the GAPS test.

**Table 9:** Numbers of children categorized as pass/fail (%) for the control children on GAPS test for the three criteria typically used in clinical and research contexts.

Control Children	GAPS Phonology 5%		
**GAPS Grammar 5%**		Pass	Fail
	Pass	30 (100)	0
	Fail	0	0
	**GAPS Phonology 10%**		
**GAPS Grammar 10%**		Pass	Fail
	Pass	29 (96.7)	1 (3.3)
	Fail	0	0
	**GAPS Phonology 15%**		
**GAPS Grammar 15%**		Pass	Fail
	Pass	28 (93.3)	2 (6.7)
	Fail	0	0

First, for the GAPS grammar subtest, 100% of children in the control group passed at the 15^th^ percentile. All controls passed the phonology screening test above the 5^th^ percentile with one child failing at the 10^th^ percentile and two scoring below the 15^th^ percentile level for their age. Overall, 100% of the controls passed the GAPS test at the 5^th^ percentile and 93.3% (29/30) of controls passed at the 10^th^ percentile, with two children identified on the phonology subtest as in need of re-test in 6 months (15^th^ percentile criterion). Thus one child was identified as in need for “referral” according to the GAPS test. Using data from the pass/fail patterns, the sensitivity, specificity and overall accuracy measures of both subtests of the GAPS were calculated using the following formulae [64]:

- Sensitivity: The number of impaired children scoring at or below the cut-off point divided by the total number of impaired children (X 100).
- Specificity: Number of non-impaired children scoring above the cut-off point divided by the total number of non-impaired children (X 100).
- Accuracy: Number of impaired children identified, added to the number of non-impaired children identified divided by the total number of impaired and non-impaired children (X100).

The sensitivity and specificity for the GAPS test combining both subtests was calculated using numbers of children failing one or both subtests. We focus on the data from children falling within the test norms (Control and Y-SLI children). The results in table 10 show that the GAPS test was 100% sensitive and specific at the 5^th^ percentile cut-off; therefore impaired vs. non-impaired children were accurately identified. Overall measures of accuracy at the 10% cut-off were also high with 98% of the children correctly identified. Although the results indicate that the 5th percentile provides the most accurate cut off point, the 10^th^ percentile is also an appropriate cut-off at which to recommend further assessment. Our results show that this cut-off level may err on the side of caution being 100% sensitive but slightly over specific with one child identified as potentially having weaknesses in phonology. Furthermore, at the 10^th^ percentile cut-off point, the sensitivity measure showed a higher correlation between the two subtests and better specificity than at the 15^th^ percentile.

**Table 10:** Percentages for the GAPS sensitivity, specificity and overall accuracy for the children within the test standardized age range (Y-SLI children n = 11 and Control children, n = 30).

Criterion	Sensitivity	Specificity	Overall accuracy
	%	%	%
	N = 11	n = 30	N = 31
**GAPS overall**			
5%	100	100	100
10%	100	96.7	98.4
15%	100	93.3	95.1
**GAPS Grammar**			
5%	72.7	100	92.7
10%	90.9	100	97.5
15%	90.9	100	97.5
**GAPS Phonology**			
5%	90.9	100	97.5
10%	90.9	96.7	93.8
15%	90.9%	93.3	92.1

The accuracy of the GAPS in distinguishing impaired from non-impaired children was further analysed using a receiver operating characteristics (ROC) analysis which produces a ROC curve and provides an overall evaluation. A combined ROC curve was generated for both subtests in order to examine the accuracy of the GAPS test as a whole. The lower percentile on either test was taken as the level of failure. In a few instances participants had passed the grammar but failed the phonology sub-test or vice versa. Percentiles were calculated separately according to whether that participant had been classified as impaired or non-impaired (0 or 1). Therefore the ROC curve represents the likelihood that either the grammar or the phonology subtest would correctly classify participants into the control or SLI groups. Tables 11 and 12 provide the ROC results showing the Area Under the Curve (AUC) statistic for the overall GAPS test, as well as the subtests individually. Y-SLI children within the test range (Table 11) and O-SLI children, above the test range (Table 12) are shown.

**Table 11:** Receiver Operating Characteristics for the GAPS Test with the Y-SLI children (Control: N = 30; Y-SLI = 11).

Test	Criterion	AUC
Overall		
	5%	1.000
	10%	.983
	15%	.967
Grammar subtest		
	5%	.864
	10%	.955
	15%	.955
Phonology-subtest		
	5%	.955
	10%	.938
	15%	.921

**Table 12:** Receiver Operating Characteristics for the GAPS test with older O-SLI children (Control: N = 30; O-SLI = 10).

Test	Criterion	AUC
Overall		
	5%	.850
	10%	.833
	15%	.917
Grammar subtest		
	5%	.850
	10%	.850
	15%	.950
Phonology subtest		
	5%	.700
	10%	.733
	15%	.717

The results indicate that the GAPS test as a whole is highly accurate in classifying impaired and non-impaired children, and both subtests may contribute to the identification of impairment. Thus, the conclusion drawn on the basis of the sensitivity and specificity percentages were supported by the ROC analysis: overall the GAPS test was highly accurate with a perfect score (AUC = 1.0) at the 5^th^ percentile and was only slightly less accurate at the 10^th^ and 15^th^ percentile (see Table 11). Sensitivity and specificity percentages for the subtests using the ROC analysis, revealed that at the 10^th^ percentile the grammar subtest was highly accurate (AUC = 0.955) as was the phonology (AUC = 0.938). Finally, we calculated the AUC for the overall test for the O-SLI group. This revealed a lower but still moderately high accuracy at the 15^th^ percentile (see Table 12).

## Discussion

This study provides data to further validate the GAPS test; a 10 minute, simple test for screening grammatical and phonological abilities that are pre-literacy skills in young children. The results demonstrated that the GAPS test shows high correlations with other tests of language and is a highly accurately screening tool for identifying children with impaired grammatical and/or phonological abilities.

Overall correlations were highly significant with the highest correlations between tests tapping similar components of language ability, for example syntactic components tapped by the CELF-RS and VATT tense marked score (*r* = 0.76). Correlations between reading and phonology skills were moderate: 0.51 for the WORD and the GAPS phonology, and 0.63 for the WORD and the CNRep. The WORD subtest may not have accurately reflected literacy skills, as the majority of the children participating were below the standardization age. This may also be because reading development is not exclusively related to phonology, but linked to many components of language and other cognitive abilities. Correlations between the GAPS subtests and the general measures of language skills (BPVS and TROG-2) ranged in strength between 0.47 for the GAPS phonology and TROG-2 and 0.71 for the GAPS grammar and BPVS. This pattern follows what might be expected due to the different language components being assessed, with the weakest correlation found between phonology and grammar. Weaker correlations were also found between the TAPS-R (a complex measure of sentence understanding) and measures of phonology (see Table 5). Correlations in this study between the GAPS test and the other standardized assessments were generally higher than those found by Gardner et al. [25]. Gardner et al. compared performance on the GAPS grammar test to the Word Structures (*r* = 0.43) and Sentence Structures (*r* = 0.52) subtest of the CELF Pre-school. In the present study, the higher correlation between the GAPS grammar subtest and the CELF-RS subtest (0.87) could be due to the two tests being more comparable in both the nature of the task and the language component being tested. There was also a stronger relationship between GAPS phonology subtest and the CNRep (r = 0.83 in this study compared to *r* = 0.67 in Gardner et al.). Similarly, the correlation between the individual subtests of the GAPS test itself was 0.84 compared to the highest correlation of 0.68 in Gardner et al. study.

These findings are consistent with previous evidence showing that phonological ability may be more closely related to the production of morphology than to the comprehension of syntax. One explanation for this is that morphological inflections can cause the phonological structure of the word to increase, for example by adding complexity to the cluster as in *“jumped”* [30]. The impact of phonological complexity (and low frequency of cluster) is particularly relevant for children with SLI when some phonotactic clusters such as *gd* or *vd,* as in *hugged, loved,* only occur in inflected words [30], [45]. The overall high correlations between the other language tests and the GAPS subtests demonstrate the validity of the GAPS test in assessing grammatical and phonological components of language across the range of abilities in the target population. This may be of particular value in the scientific community for identifying grammar and phonological abilities from impaired to high normal abilities to clarify phonotypic characteristics or potentially linking pheno-genotypic characteristics in the future.

The results demonstrate that the GAPS test has the scope to discriminate children with clinical language impairment from typically developing children: 100% of the children in the Y-SLI group performed below the level expected for their age, failing at least one subtest at the 10% cut-off level. Over 80% of the children failed both GAPS subtests at the 10^th^ percentile with 63% failing at the 5^th^ percentile, indicating that approximately half of the Y-SLI group were significantly impaired in grammar and phonology consistent with previous research findings [65]. The percentage of children failing both subtests at the 10% cut-off is higher than that found by Gardner et al. [25]: here 82.%, compared to 41% in the Gardner et al. study and this may reflect the homogeneity of this particular group of SLI children. Indeed, Ebbels [66] found that in older teenage children with SLI only half of the group showed any phonological deficits, although they did not differ on their grammatical impairment as measured by the full CELF-3, or the TAPS-R test used in this study. Our data for the O-SLI group, showing only 40% failed the phonology subtest, supports Ebbel et al. 's previous findings. There are several possible explanation as to why this occurs. On the one hand, phonology could be more receptive to treatment. On the other hand remediation of phonological problems is more likely to occur. It is also evident, at least in the UK, that directed treatment of phonology impairment is common and has a long history, but that for grammatical impairment it is relatively rare, even though such treatment has been shown to be effective [67], [68].

Although there is a need for caution when using the test with older children, this study reveals that for children between 6∶8 and 8∶11 at the 15^th^ percentile 90% of the children were identified as failing one or both subtests; a level of accuracy that remains high. These data support previous research indicating lower sensitivity outside the standardisation age, however they also suggest that standardisation at an older age level is warranted, as deficits in this age group may still be identified with this short, simple test.

One of the reasons identifying developmental language disorders is challenging is because of the heterogeneity which may be encompassed by a clinical diagnosis of SLI or dyslexia [69]. For example children with so-called Pragmatic-SLI may perform relatively well on grammar tasks [70]. Clearly, GAPS is only designed to pick up grammatical and phonological impairments and not ones in other components of language (e.g., lexical, pragmatic). The proportion of SLI children within the test norms passing both subtests in the current study was zero at the 10^th^ percentile. This may be the result of diagnostic criteria being supported for the majority of children by an independent formal statement of educational needs and therefore more consistently applied and because the SLI participants were recruited by one speech and language therapist largely on this basis. Despite this, there was some evidence of heterogeneity in this clinical group: for example, one child was identified as having a specific phonological deficit; he passed the grammar subtest at a high level, yet failed the phonology subtest at the 5^th^ percentile. However, despite this heterogeneity the GAPS test clearly differentiated the children with SLI from controls. Specificity, the results showed that only one control child failed the phonology subtest at the 10^th^ percentile and two children failed the phonology subtest at the more lenient 15^th^ percentile cut-off. This suggests that the subtests of the GAPS test probe skills that could be considered as clinical markers for SLI and/or dyslexia: syntactic and morpho-syntactic complexity and phonological structural complexity which may both be tapped in repetition tasks. We strongly emphasise that it is not the methodology per se (repetition) but the content of the test that is crucial. This is apparent if comparisons between tests, some using the same paradigm, are compared (see Table 13). The pattern of sensitivity and specificity shown in Table 13 across tests tapping phonology and grammar reflects previous studies, which have found that grammatical abilities are more accurate than phonology abilities alone in identifying children with SLI [64], [65], [71]. Botting and Conti-Ramsden [65] suggest that it is more advantageous for a measure to be over-sensitive and under-specific in the identification of impaired language, rather than for impaired children to remain unidentified. However, although for the individual child it is advantageous, such over-sensitivity has wider implications for resources and could be unnecessarily expensive to educational and health services. The results of our study suggest that the GAPS test is neither over-sensitive nor under-specific. The overall accuracy of the GAPS test as a whole is high: 100% the 5^th^ percentile and 98% at the 10^th^ percentile cut-off points. The decision as to which cut off point to choose will be a matter for the individual, health/education services or scientific criteria. However, these data provide a basis for those decisions.

**Table 13:** Comparisons of sensitivity and specificity measures of tests across three studies.

Study (classifying SLI vs. controls)	Criterion	Phonology:		Grammar:		Grammar	
		Non-word repetition		Sentence Repetition		Elicitation of Past tense Marking	
		Sensitivity	Specificity	Sensitivity	Specificity	Sensitivity	Specificity
Conti-Ramsden, 2003	16th	59%	100%			52%	100%
Botting & Conti-Ramsden, 2003	16^th^	79%	87%	90%	85%	89%	89%
GAPS sub-tests	15th	91%	93%	91%	100%		

Table Legend: Comparison of the different test content across similar and different paradigms (non-word repetition or sentence repetition) or elicited production taken from two previous studies and this study and their resulting sensitivity and specificity measures. Here the two GAPS subtest are compared separately; one under grammar and one under phonology. For measures of phonology the GAPS-phonology subtest was the most sensitive across the studies. However, the NWRep was more specific in Conti-Ramsden's 2003 study. However, as in this study, Conti-Ramsden found that 40% of the control children were incorrectly identified as “impaired”. The higher specificity of the GAPS Grammar subtest also differentiates it from the CELF-RS.

This study has provided evidence of the GAPS test’s concurrent validity, through highly significant and strong correlations between the subtests of the GAPS test and other longer tests of grammar and phonology using similar paradigms. It has also demonstrated that performance on the GAPS test accurately identified non-impaired and impaired participants, which is a crucial ethical factor in the professional use of tests [22], [27]. Validation of language tests is a cumulative process [26] and the data obtained through this study builds on previous work [25], contributing to the validation of the GAPS test. However, our use of a selected population with an over-representation of SLI with respect to the prevalence of SLI in the population at large, could have overestimated the sensitivity estimates obtained in this study. Futhermore, in our study, the experimenter was not blind to the status of the children, which could have affected the results. Ideally, testers should be blind to the status of the participant's diagnosis. Validation of a test should not be concluded with a relatively small-scale study and therefore further work by independent researchers is needed to develop this preliminary body of evidence. There are various ways this could be done. However, caution is express due to the current state of the field, which lacks a “gold-standard” test for identifying SLI. This prevents methods which simply employ another standard language test as a basis for validating and evaluating sensitivity and specificity; indeed neither test might identify those children with SLI. Thus, a clear diagnosis of SLI is required by specialists, independent of the study. This problem is illustrated by another recent study by Nash, Leavett and Childs (2011) [72] which also evaluated the GAPS test. It was based on the premise that if the evaluated test identified a different set of children as “impaired” from those identified by another test, then the evaluated test was not sensitive. However, this study appears fatally flawed as none of the children were assessed by a professional and none had a diagnosis of SLI, so we have no idea whether either test identified those children with SLI. However, based on this premise, Nash et al (2011) inappropriately concluded that GAPS has low sensitivity. Testing an unselected group with, crucially, follow-up professional assessment (of affected and unaffected children) – a step omitted in Nash et al— would provide an appropriate next step. Furthermore, a longitudinal study would provide a stringent evaluation, not currently available for any test as far as we are aware, albeit highly warranted. With such longitudinal research, the predictive validity of the GAPS test could also be explored; a common method in demonstrating the validity of screening tests [26]. Another way would be to evaluate the accuracy of GAPS in identifying grammatical impairments in other developmental disorders such as ASD or Down's syndrome. Although this study was based on results from over 50 participants, greater numbers would also be advantageous.

Finally, a qualified and experience speech and language therapist tested the children in this study. The testing by a professional speech and language therapist may also have contributed to the very high accuracy that was found. Although this preliminary study has shown that by using professional people the test provides a highly accurate screening test, further investigation is needed to evaluate the effect of assessor on overall sensitivity and specificity; i.e., professionals vs. non-professionals. The test was designed to be used by non-professionals as well as professionals making it a highly flexible screening tool.

### Conclusion

The validation data provided by this study indicates that the GAPS test is highly accurate in screening pre-school and early school-age children to identify impaired vs. non-impaired children. Scores on the GAPS were highly significantly correlated with those on tests tapping similar components of language indicating that it provides a measure of abilities across the normative range. Furthermore the impaired children had received a professional diagnosis of SLI confirming their status. We therefore suggest that the GAPS testis a realistic screening tool that may be utilized in a range of settings for clinical/educational as well as scientific purposes. Scientifically, one example could be to provide an accurate phenotypic measure of grammar for later genetic analysis. Further investigation of the concurrent and predictive validity of this quick, simple screening tool could elucidate the contribution such a test could make to the early identification and remediation of impairments to core language and pre-literacy skills. The potential impact of such tools is reflected by the huge cost of language and literacy impairments. Thereby such a scientifically based tool could make a significant difference at both the individual level and to society as a whole.

## Supporting Information

Appendix S1 Appendix S1 Individual Raw scores and age adjusted Z-scores for the children with SLI for the comprehension and expressive language tests. Key : TROG  = Test of reception of grammar-2- a test of sentence understanding [60]; BPVS  =  British picture vocabulary scales- a test of single word understanding [59]; TAPS  =  Test of active and passive sentences- Revised edition- a test of understanding reversible active and passive sentences [51]; CELF-RS =  Recalling Sentences subtest of the pre-school CELF-3 [56]; CNRep  =  The children's test of non-word repetition [57], WORD  =  Weschler objective reading dimensions [61]. VATT  =  Verb and Tense Test- an elicitation test of verb agreement (VATT-Agr) and verb past tense tense (VATT Tense) [53], GAPS-Gram  =  Grammar and phonology screening test, grammar sub-test [24], GAPS phon =  GAPS phonological subtest [24], RS =  Raw score; Z-score =  Z residual score; %  =  Percent, %ile  =  percentile; Y-SLI  =  Young-SLI children (within the standardization age-range of the GAPS test); O-SLI  =  Older-SLI children (outside the standardization age-range of the GAPS test). Y;M =  Years; Months.
(DOCX)
