# Supplementary material for: An Investigation to Validate the Grammar and Phonology Screening (GAPS) Test to Identify Children with Specific Language Impairment
Source: PLoS One. 2011 Jul 28;6(7):e22432. doi: 10.1371/journal.pone.0022432 (PMC3145645; doi:10.1371/journal.pone.0022432)
Supplement: Appendix S1 — Individual Raw scores and age adjusted Z-scores for the children with SLI for the comprehension and expressive language tests. Key : TROG = Test of reception of grammar-2- a test of sentence understanding [60]; BPVS = British picture vocabulary scales- a test of single word understanding [59]; TAPS = Test of active and passive sentences- Revised edition- a test of understanding reversible active and passive sentences [51]; CELF-RS = Recalling Sentences subtest of the pre-school CELF-3 [56]; CNRep = The children's test of non-word repetition [57], WORD = Weschler objective reading dimensions [61]. VATT = Verb and Tense Test- an elicitation test of verb agreement (VATT-Agr) and verb past tense tense (VATT Tense) [53], GAPS-Gram = Grammar and phonology screening test, grammar sub-test [24], GAPS phon = GAPS phonological subtest [24], RS = Raw score; Z-score = Z residual score; % = Percent, %ile = percentile; Y-SLI = Young-SLI children (within the standardization age-range of the GAPS test); O-SLI = Older-SLI children (outside the standardization age-range of the GAPS test). Y;M = Years; Months. (DOCX) [file pone.0022432.s001.docx]

# Appendix S1

| Group | Age | TROG | TROG | BPVS | BPVS | TAPS | TAPS | CELF-RS | CELF-RS | CNRep | CNRep | WORD | WORD | VATT | VATT | GAPS-Gram | GAPS-Gram | GAPS Phon | GAPS Phon |
| --- | --- | --- | --- | --- | --- | --- | --- | --- | --- | --- | --- | --- | --- | --- | --- | --- | --- | --- | --- |
|  | Y:M | RS | Z-score | RS | Z-score | RS | % correct | RS | Z-score | RS | Z-score | RS | Z-score | Agr  RS | Tense  RS | RS | %ile | RS | %ile |
| Y-SLI | 3;11 | 0 |  | 19 | -1.67 | 17 | 35 | 2 | -3.33 | 1 | . | 0 | . | 0 | 0 | 1 | 9 | 0 | 5 |
| Y-SLI | 3;10 | 2 |  | 36 | -0.33 | 12 | 25 | 1 | -3.33 | 0 | . | 4 | . | 0 | 0 | 0 | 5 | 0 | 5 |
| Y-SLI | 4;9 | 2 | -1.27 | 35 | -0.93 | 17 | 35 | 3 | -4.67 | 2 | -2.4 | 0 | . | 1 | 0 | 2 | 4 | 0 | 4 |
| Y-SLI | 4;10 | 0 | -1.27 | 45 | -0.27 | 25 | 52 | 2 | -4.67 | 0 | -2.4 | 2 | . | 0 | 0 | 2 | 4 | 0 | 4 |
| Y-SLI | 4;11 | 4 | -0.67 | 47 | -0.13 | 19 | 40 | 5 | -3.33 | 2 | -2.4 | 1 | . | 0 | 0 | 3 | 5 | 0 | 4 |
| Y-SLI | 5;2 | 3 | -1.4 | 40 | -0.73 | 22 | 46 | 8 | -3.33 | 0 | -2.47 | 1 | . | 2 | 0 | 4 | 8 | 0 | 4 |
| Y-SLI | 5;5 | 3 | -1.4 | 45 | -0.53 | 29 | 60 | 6 | -4 | 0 | -2.4 | 2 | . | 0 | 0 | 2 | 4 | 0 | 4 |
| Y-SLI | 6;1 | 3 | -2.33 | 44 | -1.07 | 16 | 33 | 5 | -5.33 | 1 | -2.87 | 6 | -0.67 | 0 | 0 | 0 | 0 | 0 | 0 |
| Y-SLI | 6;3 | 4 | -2.07 | 56 | -0.47 | 36 | 75 | 7 | -4.67 | 9 | -2.73 | 11 | -0.13 | 0 | 0 | 1 | 0 | 1 | 1 |
| Y-SLI | 6;5 | 14 | 1.07 | 56 | -0.47 | 35 | 73 | 25 | -0.67 | 9 | -2.73 | 7 | -0.87 | 13 | 4 | 10 | 40 | 3 | 3 |
| Y-SLI | 6;6 | 4 | -2.53 | 59 | -0.47 | 23 | 48 | 9 | -4.67 | 11 | -2.4 | 3 | -0.87 | 0 | 0 | 5 | 0 | 7 | 65 |
|  |  |  |  |  |  |  |  |  |  |  |  |  |  |  |  |  |  |  |  |
| O-SLI | 6;9 | 3 | -2.8 | 53 | -0.87 | 39 | 81 | 16 | -2.67 | 18 | -1.13 | 5 | -1.27 | 2 | 0 | 5 | 0 | 5 | 17 |
| O-SLI | 7;2 | 6 | -2.33 | 63 | -0.53 | 25 | 52 | 9 |  | 22 | -1.13 | 20 | . | 0 | 0 | 7 | 0 | 5 | 17 |
| O-SLI | 7;2 | 8 | -1.73 | 57 | -0.8 | 35 | 73 | 11 |  | 23 | -0.93 | 8 | . | 0 | 0 | 10 | 40 | 8 | 100 |
| O-SLI | 7;4 | 4 | -3 | 51 | -1.2 | 21 | 44 | 6 |  | 10 | -3.2 | 12 | . | 0 | 0 | 2 | 0 | 3 | 3 |
| O-SLI | 7;4 | 6 | -2.33 | 48 | -1.4 | 18 | 38 | 9 |  | 7 | -3.33 | 8 | . | 0 | 0 | 3 | 0 | 1 | 1 |
| O-SLI | 7;6 | 2 | -3 | 78 | 0 | 31 | 65 | 16 |  | 12 | -2.87 | 15 | . | 0 | 0 | 4 | 0 | 3 | 3 |
| O-SLI | 8;2 | 14 | -2.2 | 65 | -0.8 | 34 | 71 | 15 |  | 14 | -3.6 | 8 | . | 8 | 5 | 6 | 0 | 4 | 7 |
| O-SLI | 8;4 | 12 | -1 | 66 | -1.07 | 34 | 71 | 22 |  | 23 | -2.53 | 11 | . | 5 | 3 | 9 | 13 | 7 | 65 |
| O-SLI | 8;10 | 6 | -2.8 | 50 | -2.13 | 15 | 31 | 12 |  | 12 | -3.6 | 15 | . | 2 | 3 | 2 | 0 | 3 | 3 |
| O-SLI | 8;11 | 14 | -0.33 | 76 | -0.8 | 30 | 63 | 29 |  | 25 | -2 | 36 | . | 18 | 8 | 9 | 13 | 8 | 100 |
